# Deciphering the Efficacy of β-Lactams in the Face of Metallo-β-Lactamase-Derived Resistance in Enterobacterales: Supraphysiologic Zinc in the Broth Is the Culprit

**DOI:** 10.1093/ofid/ofae228

**Published:** 2024-04-23

**Authors:** Kamilia Abdelraouf, Christian M Gill, Matthew Gethers, Giusy Tiseo, Simona Barnini, Marco Falcone, Francesco Menichetti, David P Nicolau

**Affiliations:** Center for Anti-Infective Research & Development, Hartford Hospital, Hartford, Connecticut, USA; Center for Anti-Infective Research & Development, Hartford Hospital, Hartford, Connecticut, USA; Center for Anti-Infective Research & Development, Hartford Hospital, Hartford, Connecticut, USA; Infectious Diseases Unit, Department of Clinical and Experimental Medicine, Azienda Ospedaliera Universitaria Pisana, University of Pisa, Pisa, Italy; Microbiology Unit, Azienda Ospedaliera Universitaria Pisana, Pisa, Italy; Infectious Diseases Unit, Department of Clinical and Experimental Medicine, Azienda Ospedaliera Universitaria Pisana, University of Pisa, Pisa, Italy; Infectious Diseases Unit, Department of Clinical and Experimental Medicine, Azienda Ospedaliera Universitaria Pisana, University of Pisa, Pisa, Italy; Center for Anti-Infective Research & Development, Hartford Hospital, Hartford, Connecticut, USA

**Keywords:** bloodstream infections, carbapenem, *Klebsiella pneumoniae*, NDM, zinc

## Abstract

**Background:**

*In vitro–in vivo* discordance in β-lactams’ activities against metallo-ß-lactamase (MBL)-producing Enterobacterales has been described. We aimed to assess whether this discordance is attributed to the supra-physiologic zinc concentration in *in vitro* testing media.

**Methods:**

A clinical and microbiological observational study of patients with bloodstream infections due to New Delhi metallo-ß-lactamase-producing *Klebsiella pneumoniae* was performed. Outcomes of patients treated empirically with non-MBL-active β-lactam therapy (carbapenems and ceftazidime/avibactam) and MBL-active β-lactam therapy (ceftazidime/avibactam + aztreonam) were documented. The patients’ isolates were used to induce septicemia in mice, and survival upon meropenem treatment was recorded. Meropenem minimum inhibitory concentrations (MICs) were determined in standard media and in the presence of physiological zinc concentrations.

**Results:**

Twenty-nine patients receiving empiric non-MBL-active β-lactams (median duration, 4 days) were compared with 29 receiving MBL-active β-lactams. The 14-day mortality rates were 21% and 14%, respectively. In the murine septicemia model, meropenem treatment resulted in protection from mortality (*P* < .0001). Meropenem MICs in the physiologic zinc concentration broth were 1- to >16-fold lower vs MICs in zinc-unadjusted broth (≥64 mg/L).

**Conclusions:**

Our data provide foundational support to establish pharmacokinetic/pharmacodynamic relationships using MICs derived in physiologic zinc concentration, which may better predict β-lactam therapy outcome.

Metallo-β-lactamases (MBLs), such as New Delhi metallo-ß-lactamase (NDM), VIM, and IMP types, are broad-spectrum β-lactamases that have the capability to hydrolyze the majority of the commercially available β-lactam antibiotics. Studies that assess the clinical outcome of infections due to MBL-producing Enterobacterales are currently scarce. A few published reports have previously described positive outcomes among patients treated with β-lactam-based therapies, despite the perceived *in vitro* resistance [1–4], which may cast doubt on the suitability of current *in vitro* tests to reliably guide therapy. However, these reports were mostly case reports or series and thus were associated with high risk of bias. Additionally, the limited numbers of patients identified with MBL-producing Enterobacterales infections that were empirically treated with β-lactam-based regimens preclude the statistical assessment of the therapy outcome.

The *in vivo* efficacy of β-lactam agents including ceftazidime/avibactam and carbapenems against MBL-producing Enterobacterales infections in murine infection models supports the argument that *in vitro–in vivo* discordance exists when the agents are dosed to attain clinically achievable exposures [5–7]. Systematic assessments of the *in vitro–in vivo* discordance in meropenem activity against MBL-producing Enterobacterales have revealed a major flaw; the zinc concentration in conventional culture media such as cation-adjusted Mueller Hinton Broth (CAMHB) utilized in broth microdilution is much higher than physiologic zinc concentrations, particularly at infection sites [5]. Indeed, susceptibility testing systems in many respects may fail to replicate the physiological factors that exist during host–pathogen interactions, which can significantly impact results and, consequently, the ability of the test to predict the outcome of antibiotic therapy [8, 9]. Consistent with our preclinical observations, a recently published study that compared the outcomes of patients with infections due to MBL- vs non-MBL-carbapenemase-producing *Escherichia coli* reported higher mortality among patients with non-MBL isolates, despite the fact that only 16% of the patients with MBL-producing *E. coli* received an active antibiotic according to susceptibilities reported by the local laboratory. Interestingly, ∼50% of the MBL-producing *E. coli* patients received at least 1 dose of carbapenem [10]. Of note, infections with MBL-producing *E. coli* were more commonly from urine sources, and the patients have been shown to have lower severity of illness compared with non-MBL infections [10].

Efforts to adjust *in vitro* susceptibility testing media to better mimic physiological conditions have led to more clinically meaningful susceptibility results, which are particularly crucial for certain bug/drug combinations [11, 12]. The impact of the discrepancy in zinc concentrations is significant for MBL-producing organisms because all subtypes of clinically important MBLs utilize 1 or 2 zinc atoms in their active site to facilitate bicyclic β-lactam ring hydrolysis [13]. Thus, under the zinc-poor conditions that exist at the site of infection, MBLs appear to lose their β-lactam hydrolytic capability, as evidenced by marked β-lactam *in vivo* activity. On the contrary, MBL-producing isolates demonstrate high-level *in vitro* resistance to all β-lactams when tested using standard zinc-rich media. The supra-physiological zinc content in CAMHB can distort susceptibility testing and the potential clinical utility of most β-lactams [14].

In this study, we evaluated the outcome of ß-lactam therapy, mainly carbapenems, in the setting of NDM-producing *Klebsiella pneumoniae* bacteremia through a comprehensive approach that encompassed the utilization of clinical bacterial strains in a translational murine infection model. We next modified the minimum inhibitory concentration (MIC) testing methodology to provide MIC values predictive of the *in vivo* response to carbapenems against MBL-producing Enterobacterales through adjustment of the zinc concentration in CAMHB to reflect physiological concentrations.

## METHODS

### Study Design

This was an observational study including isolates from patients with MBL-producing Enterobacterales infections identified from the Azienda Ospedaliero Universitaria Pisana (Pisa, Italy) prospectively collected registry for patients infected with carbapenem-resistant Enterobacterales in 9 hospitals in the northwestern area of Tuscany.

Patients with mono-microbial bloodstream infection originating from any source due to NDM-harboring *K. pneumoniae* were identified from the registry during the NDM outbreak in Tuscany, Northern Italy, between 2018 and 2021 [15]. Patients who received empiric therapy with ceftazidime/avibactam plus aztreonam (MBL-active) or either meropenem, imipenem, or ceftazidime/avibactam without aztreonam (non-MBL-active) were eligible. Patients who received empiric antibiotics that can potentially have activity against the MBL producer, such as colistin, were excluded. Exclusion criteria also included the unavailability of the infecting isolate or the receipt of empiric therapy that did not fall under the 2 test groups. Empiric therapy was defined as therapy initiated before the result of the microbiologic procedures identifying that positive blood culture grew NDM-harboring *K. pneumoniae*. Cohort designation was based on the empiric therapy being either MBL-active or non-MBL-active. Targeted therapy was the therapy that was initiated in response to the microbiologic findings at the discretion of the treating clinicians.

### Clinical Variables and Outcomes

Baseline demographics, comorbidities, and infection-related risk factors were abstracted from the database. Continuous variables were reported as medians and interquartile ranges (IQRs). Categorical data were reported as frequency distributions. The primary outcome was 14-day all-cause mortality. Thirty-day all-cause mortality and microbiological failure were assessed as secondary outcomes. Due to the relatively small sample size and exploratory nature of the analysis, inferential statistics were not performed.

### Isolates and MICs in Conventional CAMHB

Isolates were collected from 58 patients. Five clinical Enterobacterales isolates were used as controls; KP 558 and KP 561 were meropenem-resistant, harboring KPC-3 and OXA-48 enzymes, respectively, while EC 471, KO 92, and KP 1108 were meropenem-susceptible [16]. Plazomicin, as a non-ß-lactam control, was incorporated into both the *in vitro* and *in vivo* studies. All MIC testing was performed in triplicate using Clinical and Laboratory Standards Institute (CLSI) broth microdilution methods [17, 18] and a single lot of BBL Mueller Hinton II Broth (Cation-Adjusted, lot 0286591), as well as analytical grades of plazomicin sulphate (Achaogen, South San Francisco, CA, USA, lot EX5-248) and meropenem trihydrate (Sigma-Aldrich, St. Louis, MO, USA, lot LRAC5653).

### *In Vivo* Validation in a Murine Septicemia Model

Specific pathogen-free, female ICR mice (20–22 g) were obtained from Charles River Laboratories, Inc. (Wilmington, MA, USA). Mice were provided food and water ad libitum and allowed to acclimate for a minimum of 48 hours before commencement of experimentation. The protocol was approved by the Institutional Animal Care and Use Committee at Hartford Hospital (Assurance #A3185-01). Septicemia with the NDM-producing *K. pneumoniae* from patients (n = 58) and control strains (n = 5) was produced by intraperitoneal inoculation [16]. Based on an a priori *in vivo* growth trial (data not shown), the starting inoculum was adjusted for each isolate so that the initial spleens’ bacterial densities ranged from 10^3^ to 10^4^ cfu/spleen, and mortality among the sham control-treated mice was at least 80% by 24 hours. Following inoculation, mice were randomized into 1 of 4 groups: 0 hours controls (n = 6 mice per isolate), saline treatment (controls, n = 10 mice per isolate), meropenem treatment (n = 10 mice per isolate), or plazomicin treatment (n = 10 mice per isolate). One hour after inoculation, the 0 hours controls for each isolate were euthanized before aseptic spleen harvest. Spleens were homogenized in normal saline, and serial dilutions of the homogenates were plated on Trypticase Soy Agar plates with 5% sheep blood (Becton, Dickinson & Co., Sparks, MD, USA) for cfu determination to confirm the establishment of septicemia. Treatments were initiated 1 hour after inoculation and continued for 48 hours via subcutaneous injection. Meropenem vials (Fresenius Kabi, Lake Zurich, IL, USA, lot 4A2OE14) and plazomicin analytical grade were used. Meropenem treatment groups received a previously established murine regimen that provided an exposure similar to that achieved in humans following the administration of 2 g over a 3-hour infusion every 8 hours [16, 19]. Plazomicin treatment groups received a previously established murine regimen that provided an exposure similar to that achieved in human subjects following the administration of 15 mg/kg over a 0.5-hour infusion every 24 hours [16, 20]. Meropenem efficacy was assessed against all the clinical MBL-producing isolates and the 5 non-MBL-producing isolates, while plazomicin efficacy was examined against 10 plazomicin-susceptible and 10 plazomicin-resistant clinical MBL-producing isolates.

Mortality was assessed at least every 6 hours for 96 hours. Time of mortality was recorded, and the spleens were harvested from the mice that were euthanized upon the loss of righting reflex or found dead during the observation times and processed for cfu determination. At 96 hours, all surviving animals were euthanized, and the spleens were harvested for cfu determination. The staff members performing cfu determination were blinded to the treatments of the mouse groups.

Efficacy was calculated for each isolate as the change in spleens’ bacterial densities at the end point compared with the numbers in the 0 hours controls. The rate and extent of mortality were recorded and assessed. Survival was compared between groups using Kaplan-Meier survival analysis and the log-rank test. Statistical significance was established at *P* ≤ .05.

### Zinc Plasma Concentrations and Protein Binding

Determination of zinc protein binding was conducted on plasma from infected mice using a previously described ex vivo ultrafiltration methodology in triplicate [21]. The human zinc protein binding was also examined for 3 healthy human volunteers. Blood samples (5 mL) were collected in a 10-mL Monoject tube containing K2 EDTA and centrifuged at 2000 × *g* for 10 minutes at 4°C to obtain separated plasma. Plasma from each volunteer constituted 1 replicate. Separation of the ultrafiltrates were performed, and the total and unbound zinc concentrations were measured for murine and human samples using ICP-MS [5]. Zinc protein binding percentages were calculated by dividing the concentration in the ultrafiltrate by the total plasma concentration and subtracting from 100 (%).

### Susceptibility Testing in Physiologic Zinc Concentration in CAMHB

The purpose of this section was to determine the MIC of the isolates in the presence of physiological zinc concentrations. As such, the physiologic zinc concentration in CAMHB was prepared using Chelex for removal of Zn^2+^ followed by supplementation to that of physiological unbound concentrations in murine and human plasma (adapted from Hackel et al.) [12]. Briefly, Chelex 100 (Sigma-Aldrich, Saint Louis, MO, USA) was added (10% w/v) to autoclaved CAMHB (Becton, Dickinson and Company, Franklin Lakes, NJ, USA) to remove polyvalent cationic metal ions. The mixture was stirred at room temperature for 2 hours and sterilized by filtration to remove the Chelex. Next, calcium (20–25 mg/L as Ca^2+^), magnesium (10–12.5 mg/L as Mg^2+^), and iron (0.5–1 mg/L as Fe^3+^) were added per CLSI recommendations to replace cations, while zinc was adjusted to the physiological unbound concentrations in the murine and human plasma as Zn^2+^ (55 ng/mL). Finally, the broth pH was adjusted to 7.3 with hydrochloric acid and sterile-filtered.

Zinc concentrations were assayed by ICP-MS for the CAMHB before and after zinc adjustment [5]. Meropenem and plazomicin MICs were determined in triplicate in the physiologic zinc concentration in CAMHB.

## RESULTS

### Clinical Variables and Outcomes

Twenty-nine patients were identified in each of the MBL-active and non-MBL-active groups.

Among the non-MBL-active group, 24 (83%) patients received meropenem. The median duration of non-MBL-active therapy (IQR) was 4 (3–5) days, after which patients were switched to definitive therapies based on susceptibility testing results. Supplementary Table 1 shows the empiric and definitive therapies received by the patients in the non-MBL-active group. Among patients in the non-MBL-active group, only 5 (17.2%) continued on meropenem, while 12 (41.4%) were shifted to ceftazidime/avibactam plus aztreonam, 8 (27.6%) to colistin-containing regimens, and 4 (13.8%) to other regimens (eg, tigecycline ± meropenem ± fosfomycin or fosfomycin alone) within a median of 4 days from blood culture collection. Patients in the MBL-active group received ceftazidime-avibactam plus aztreonam as empiric and definitive therapies.

The majority of the baseline characteristics were balanced between the groups; however, a higher percentage of the MBL-active group was in the intensive care unit and had a longer hospital stay (Table 1). Both patient groups had high incidence of comorbidities, namely solid tumor malignancy, cardiovascular disease, and diabetes. Nevertheless, the incidence of all assessed comorbidities and Charlson comorbidity index values were similar between patient groups (Table 1). Infection-related characteristics are shown in Table 2. Patients belonging to the MBL-active group were less likely to have urinary tract infection as the origin of bacteremia. Median Sequential Organ Failure Assessment (SOFA) scores were also higher in the MBL-active group; nevertheless, the IQRs overlapped, while no differences in the achievement of source control were found. Among the patients belonging to the MBL-active and non-MBL-active groups, the 14-day mortality rates were 14% and 21%, respectively, while the 30-day mortality rates were 31% and 38%, respectively. Microbiologic failure was rare: 14% and 3%, respectively.

**Table 1.** Baseline Characteristics and Comorbidities

Characteristic	MBL-Active Group	Non-MBL-Active Group
Total patients	29	29
Sex		
Male, No. (%)	21 (72)	21 (72)
Age, y		
Median (IQR)	73 (67–79)	72 (65–78)
Body weight, kg		
Median (IQR)	72 (62–79)	72 (67–76)
Height, cm		
Median (IQR)	170 (162–180)	172 (167–177)
Body mass index, kg/m^2^		
Median (IQR)	24 (21–28)	24 (23–26)
Hospital length of stay, d		
Median (IQR)	48 (27–76)	31 (15–62)
Unit of admission, No. (%)		
Medical	6 (21)	18 (62)
Surgical	3 (10)	3 (10)
Intensive care unit	20 (69)	8 (28)
Year of admission		
2018	0	7 (24)
2019	14 (48)	19 (66)
2020	13 (45)	0
2021	2 (7)	3 (10)
Charlson Comorbidity Index		
Median (IQR)	4 (2–5)	5 (3–7)
HIV	1 (3)	0
Neutropenia	1 (3)	2 (7)
Solid tumor malignancy	14 (48)	14 (48)
Hematologic malignancy	1 (3)	2 (7)
Diabetes	11 (38)	10 (34)
Solid organ transplant	1 (3)	0 (0)
Chronic obstructive pulmonary disease	4 (14)	9 (31)
Chronic liver failure	2 (7)	1 (3)
Cardiovascular disease	17 (59)	15 (52)
Chronic kidney disease	4 (14)	6 (21)

Abbreviation: IQR, interquartile range.

**Table 2.** Infection-Related Characteristics

Infection Characteristics, No. (%)	MBL-Active Group	Non-MBL-Active Group
Site of infection		
BSI	29 (100)	29 (100)
Infecting organism		
*Klebsiella pneumoniae*	29 (100)	29 (100)
Carbapenemase gene		
NDM	29 (100)	29 (100)
Source of infection		
Central line–associated bloodstream infection	8 (28)	5 (17)
Pneumonia	8 (28)	3 (10)
Skin/skin structure	2 (7)	3 (10)
Urinary tract infection	4 (14)	10 (34)
Intra-abdominal infection	3 (10)	1 (3)
Unknown	4 (14)	7 (24)
Source control obtained	14 (48)	14 (48)
APACHE II score (n = 40 of 58)		
Median (IQR)	17 (13–23)	17 (11–20)
SOFA score		
Median (IQR)	6 (4–8)	4 (4–6)
Categorization at time of infection		
Sepsis	29 (100)	29 (100)
Septic shock	11 (38)	12 (41)
Mechanical ventilation	15 (52)	9 (31)
Acute kidney injury	5 (17)	12 (41)
Continuous renal replacement therapy	3 (10)	1 (3)
Extracorporeal membrane oxygenation	1 (3)	0
Initial lactate		
Median (IQR)	2.1 (1.5–4.3)	2.4 (1.7–3.5)
Initial WBC		
Median (IQR)	13 (10–18)	12 (8–16)
WBC >12 000, No. (%)	18 (62)	17 (59)
Initial CRP		
Median (IQR)	19 (11–25)	14 (10–35)
Initial procalcitonin		
Median (IQR)	3.7 (0.45–44.05)	9.5 (1.88–41.03)
Procalcitonin value >0.5, No. (%)	19 (76)	23 (82)
Fever at sepsis onset	22 (76)	11 (38)

Abbreviations: APACHE II, Acute Physiology and Chronic Health Evaluation II score; BSI, bloodstream infection; CRP, C-reactive protein; IQR, interquartile range; MBL, metallo-ß-lactamase; NDM, New Delhi metallo-ß-lactamase; SOFA, Sequential Organ Failure Assessment; WBC, white blood cell.

### MICs in Conventional CAMHB

**Figure 1.**
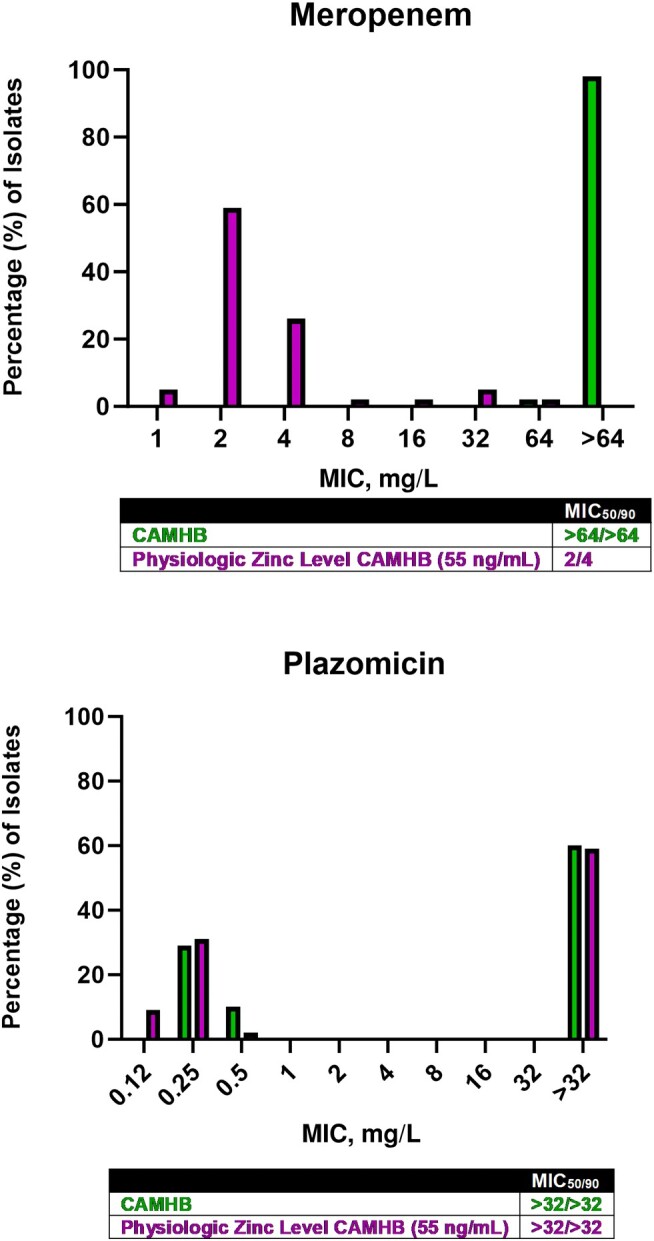
The distribution of modal meropenem (top) and plazomicin (bottom) MICs of the 58 clinical NDM-producing *K. pneumoniae* isolates in conventional and physiologic zinc concentration in CAMHB. Abbreviations: CAMHB, cation-adjusted Mueller Hinton Broth; MIC, minimum inhibitory concentration; NDM, New Delhi metallo-ß-lactamase.

The distributions of MICs in CAMHB are shown in Figure 1. Among the clinical isolates, ∼40% were plazomicin-susceptible. All meropenem MICs were ≥64 mg/L. Among the non-MBL-producing control isolates, isolates harboring serine carbapenemase genes were meropenem-resistant, while isolates lacking carbapenemase expression were susceptible (Table 3).

**Table 3.** The Modal Meropenem and Plazomicin MICs of the Clinical Non-NDM-Producing *K. pneumoniae* Isolates in Conventional CAMHB and Physiologic Zinc Concentration in CAMHB

Organism ID	β-Lactamase	MIC in CAMHB, mg/L		MIC in Physiologic Zinc Concentration in CAMHB, mg/L	
		Meropenem	Plazomicin	Meropenem	Plazomicin
KP 1108	None	≤0.06	0.5	≤0.06	0.25
KP 558	TEM-1, SHV-11, KPC-3	>64	4	>64	2
EC 471	TEM-1, intrinsic AmpC	≤0.06	4	≤0.06	4
KO 92	OXY-6–4	≤0.06	4	≤0.06	2
KP 561	TEM-1, SHV-11, CTX-M-15, OXA-48, OXA-1, OXA-30	>64	8	>64	8

Abbreviations: CAMHB, cation-adjusted Mueller Hinton Broth; EC, *Escherichia coli*; KO, *Klebsiella oxytoca*; KP, *Klebsiella pneumoniae*; MIC, minimum inhibitory concentration; NDM, New Delhi metallo-ß-lactamase.

### Activity in the Septicemia Model

**Figure 2.**
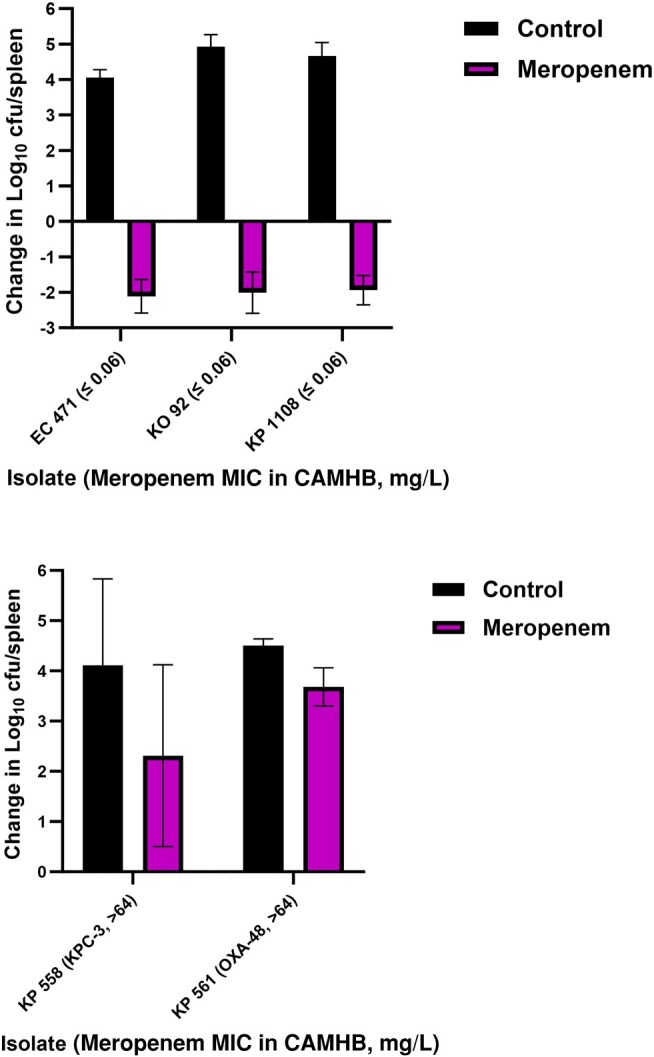
Spleens’ bacterial densities among saline controls and meropenem-treated groups infected with the 3 non-carbapenemase-producing control Enterobacterales isolates (top) and the 2 serine carbapenemase-producing control *K. pneumoniae* isolates (bottom). Abbreviations: CAMHB, cation-adjusted Mueller Hinton Broth; MIC, minimum inhibitory concentration.

**Figure 3.**
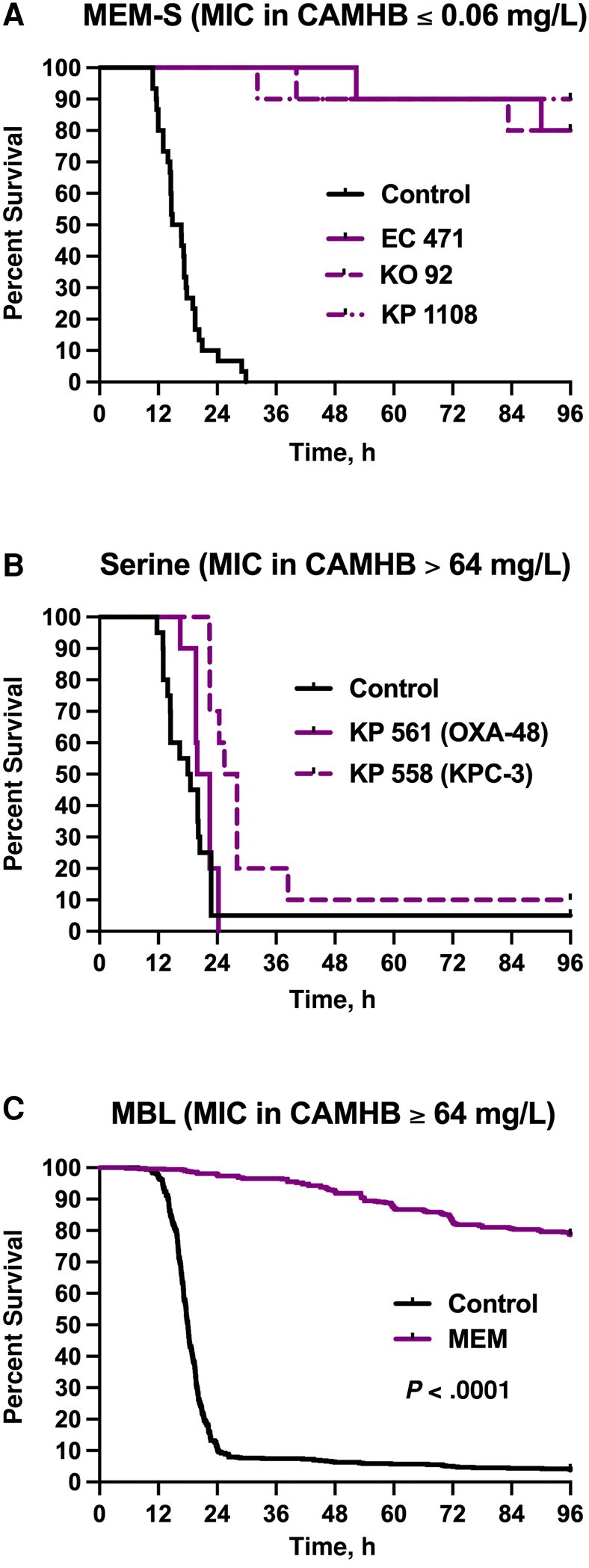
Survival curves for saline controls (black) and mice treated with meropenem (purple) and infected with (*A*) the 3 non-carbapenemase-producing control Enterobacterales isolates, (*B*) the 2 serine carbapenemase-producing control *K. pneumoniae* isolates, (*C*) the 58 clinical NDM-producing *K. pneumoniae* (pooled data). MEM-S: n = 30 mice for saline controls and 10 for each meropenem group. Serine: n = 20 mice for saline controls and 10 for each meropenem group. MBL: n = 576 mice for saline controls and 580 for the meropenem group. Abbreviations: CAMHB, cation-adjusted Mueller Hinton Broth; MBL, metallo-ß-lactamase; MIC, minimum inhibitory concentration; MEM-S, meropenem-susceptible; NDM, New Delhi metallo-ß-lactamase.

**Figure 4.**
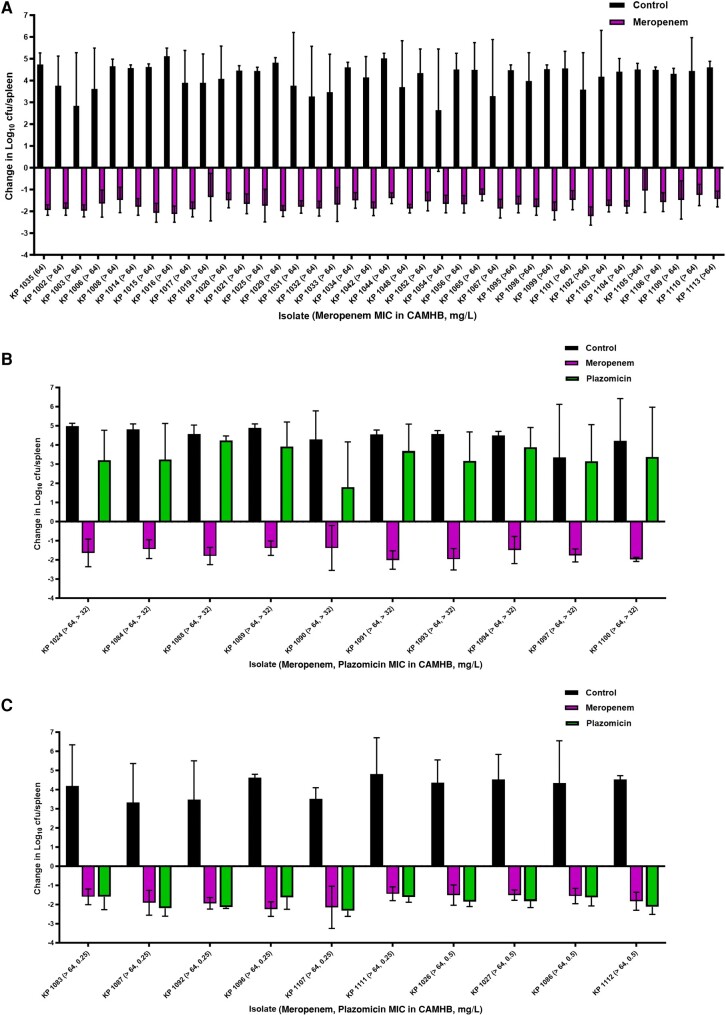
Spleens’ bacterial densities among (*A*) saline controls and meropenem-treated mice infected with 38 NDM-producing *K. pneumoniae*, (*B*) saline controls, meropenem- and plazomicin-treated mice infected with 10 plazomicin-resistant NDM-producing *K. pneumoniae*, (*C*) saline controls, meropenem- and plazomicin-treated mice infected with 10 plazomicin-susceptible NDM-producing *K. pneumoniae.* Abbreviations: CAMHB, cation-adjusted Mueller Hinton Broth; MEM, meropenem; MIC, minimum inhibitory concentration; NDM, New Delhi metallo-ß-lactamase; PLZ, plazomicin.

**Figure 5.**
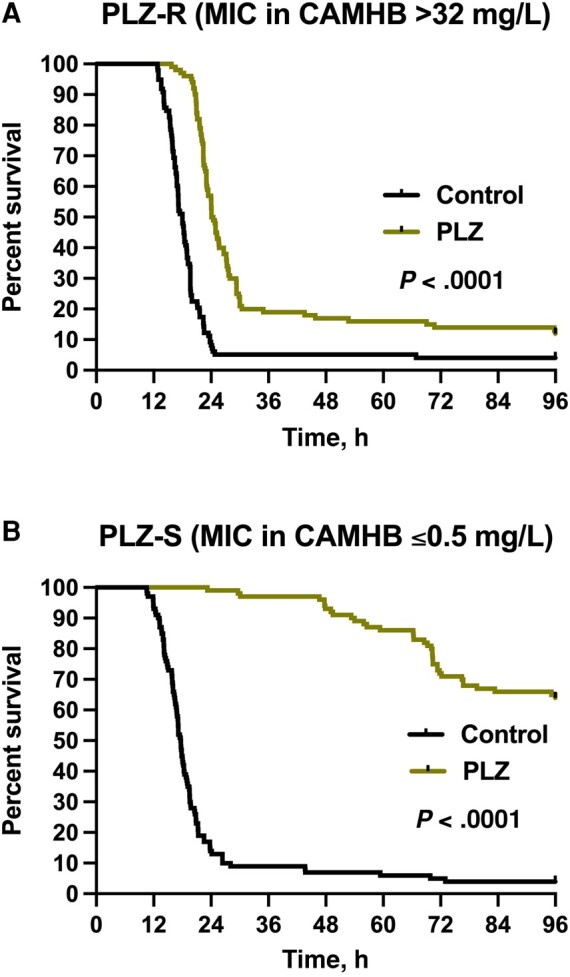
Pooled survival curves for the saline controls (black) and plazomicin-treated mice (green) infected with (*A*) the 10 plazomicin-resistant NDM-producing *K. pneumoniae*, (*B*) the 10 plazomicin-susceptible NDM-producing *K. pneumoniae.* PLZ-R: n = 98 mice for saline controls, 100 for plazomicin group. PLZ-S: n = 100 mice for each of saline controls and plazomicin groups. Abbreviations: CAMHB, cation-adjusted Mueller Hinton Broth; MIC, minimum inhibitory concentration; NDM, New Delhi metallo-ß-lactamase; PLZ, plazomicin.

Among mice infected with the non-MBL-producing isolates, meropenem *in vivo* efficacy as measured by mouse survival and changes in spleens’ bacterial densities relative to baseline were consistent with the isolates’ phenotypes (Figures 2*A* and *B* and 3*A* and *B*). On the contrary, aggregated data from all 58 NDM-harboring clinical isolates showed 78.6% survival upon meropenem treatment despite the reported *in vitro* resistance using conventional broth testing methods (Figure 3*C*), and bacterial kill was detected in the spleens (Figure 4*A*–*C*). As for the plazomicin-treated mice, mouse survival and bacterial density changes were consistent with the isolates’ plazomicin susceptibilities (Figures 4*B* and *C* and 5*A* and *B*). Against the plazomicin-susceptible isolates, meropenem treatment was superior to plazomicin and resulted in significantly enhanced survival (*P* = .0049).

### Zinc Plasma Concentrations and Protein Binding

Zinc was highly bound to plasma proteins; the average percentage of zinc protein binding in infected mice and healthy volunteers’ plasma was 95.63% ± 1.23% and 89.38% ± 7.33%, respectively (Table 4). The concentration of unbound zinc was relatively less variable in infected mouse plasma compared with healthy volunteers’—41.25 ± 9.82 ng/mL and 69.16 ± 37.59 ng/mL, respectively—but the ranges of concentrations in the 2 matrices overlapped: 33.53–52.31 and 36.97–110.48 ng/mL, respectively.

**Table 4.** **Zinc Concentrations in Infected Mice and Healthy Volunteers**’ **Plasma as Well as the Percent Protein Binding of Zinc in Each Matrix**

		Replicate 1^a^	Replicate 2	Replicate 3	Average ± SD
Infected mice	Total zinc in plasma, ng/mL	906.57	990.50	958.59	951.89 ± 42.36
	Free zinc in plasma, ng/mL	52.31	37.92	33.53	41.25 ± 9.82
	% bound	94.23	96.17	96.50	95.63 ± 1.23
		Volunteer 1	Volunteer 2	Volunteer 3	Average ± SD
Healthy volunteers	Total zinc in plasma, ng/mL	683.02	805.83	581.24	690.03 ± 112.46
	Free zinc in plasma, ng/mL	36.97	60.04	110.48	69.16 ± 37.59
	% bound	94.59	92.55	80.99	89.38 ± 7.33

^a^Each replicate constitutes pooled plasma from 5 infected mice.

### Susceptibility Testing in Physiologic Zinc Concentration in CAMHB

The results from the zinc assays for the CAMHB before and after treatment with Chelex as well as following zinc concentration adjustment to the physiologic concentration are shown in Supplementary Table 2. The final zinc concentration following adjustment was within the variabilities of the unbound zinc values in mouse and human plasma and approximated the average value (55 ng/mL). Importantly, the zinc concentration in the conventional CAMHB was consistently >1000 ng/mL, that is, >15-fold higher than the average plasma unbound zinc concentration.

Meropenem MICs in the physiologic zinc concentration in CAMHB were consistent with the genotypic characterization of the non-MBL-producing isolates; isolates harboring serine carbapenemase genes were meropenem-resistant, while isolates lacking carbapenemase expression were susceptible (Table 3). Zinc adjustment had no impact on the control isolates’ susceptibility to meropenem or plazomicin; MICs in the physiologic zinc concentration broths were similar to or within a 2-fold dilution of the values in conventional CAMHB. Among the NDM-harboring clinical isolates, the modal plazomicin MICs of both plazomicin-resistant and plazomicin-susceptible isolates were likewise unaffected by zinc adjustment. Conversely, the modal meropenem MICs were substantially lowered in response to zinc adjustment, rendering the MIC_50/90_ 2/4 mg/L (Figure 1).

## DISCUSSION

Prompt therapy with active antimicrobials is the mainstay of good outcomes for serious bacterial infections. Indeed, across numerous infection types and bacterial pathogens including carbapenem-resistant Enterobacterales, delays in time to active antimicrobial therapy have been associated with increased mortality [22, 23]. Specific to bacteremia due to KPC-producing Enterobacterales, Falcone and colleagues reported that patients who received *in vitro–*active therapy within 24 hours had the lowest 30-day mortality rate (29.1%) [24], which is comparable to the 30-day mortality seen in the present study among patients who received MBL-active or non-MBL-active empiric therapy (31% and 38%, respectively). Patients who had appropriate therapy delayed ≥72 hours had 30-day mortality occur in 66.7% [24]. In the present study, patients in the non-MBL-active group were switched to *in vitro* active definitive therapy (after a median of 4 days), which could have impacted the overall mortality rates. Nevertheless, the proportion of patients in whom mortality occurred was considerably lower than the rates reported in the literature in response to inappropriate empiric therapy for similar durations. Despite the limited number of included patients, which cannot lead to conclusions about therapeutic approach, these observations may highlight challenges about the definition of inactive therapy based on conventional MIC determination.

Using the murine septicemia model, data from the non-MBL-producing Enterobacterales isolates provided validation for the model as the clinical meropenem exposure utilized in the study produced *in vivo* activity concordant with the phenotypic and genotypic profiles of the meropenem-susceptible and meropenem-resistant control isolates. Plazomicin was selected as a non-β-lactam control because unlike other aminoglycosides, plazomicin retains activity against >50% of NDM-producing *K. pneumoniae* contingent on the absence of *armA* or *rmtC* methylase genes [25]. The *in vivo* activity of plazomicin against the clinical MBL producers was concordant with the phenotypic profile, providing further evidence of the suitability of the model to discriminate between effective and ineffective therapy. On the other hand, the marked meropenem *in vivo* activity against the clinical MBL producers in the murine septicemia model did not correspond with the elevated MICs generated in CAMHB; the meropenem plasma concentrations achieved were substantially lower than the observed MICs throughout the dosing interval, resulting in %*f*T > MIC = 0 [16]. Earlier assessments of susceptibility of Enterobacterales strains harboring various MBL genes (NDM, VIN, and IMP subtypes) in media treated with EDTA or Chelex to remove zinc showed multifold reductions in β-lactam agent MICs and no alteration in MICs to non-β-lactams relative to conventional CAMHB. Nevertheless, these assessments were mostly proof-of-concept studies as the extent of zinc depletion was not controlled [5, 14]. The focus of the next phase of the study was thus to identify the physiologic zinc concentrations, then replicate these concentrations in the testing media. The low meropenem MICs of the MBL producers in the physiologic zinc concentration in CAMHB appeared concordant with the meropenem efficacy observed *in vivo* in the septicemia model. The meropenem regimen utilized *in vivo* (2 g q8h over 3 hours infusion) provided a *f*T > MIC that exceeded the pharmacokinetic/pharmacodynamic (PK/PD) target (40%) against isolates with meropenem MICs ≤16 mg/L [16, 26] and %*f*T > MIC >90% against isolates with MICs ≤4 mg/L, which would suggest success against isolates with MICs below each threshold. Likewise, the meropenem MICs in physiologic zinc concentrations better explained the patients’ survival in the context of classic PK/PD principles considering that the majority of the patients were administered meropenem as prolonged infusion. Indeed, the zinc concentrations in the patients’ plasma are not known and are possibly along a spectrum, which likely accounts for the variability in clinical outcomes as an average free zinc from the healthy volunteers was utilized for MIC testing. Like all non-ß-lactam antibiotic classes, aminoglycoside activity is not known to be zinc-dependent; thus plazomicin MICs were not altered by zinc adjustment. Additional studies are required to assess the variability in unbound zinc concentrations among a larger cohort including in patients with active infection. Additionally, zinc concentrations in different infection sites (eg, epithelial lining fluid and urine) should be assessed. These assessments can play an important role in modifying the *in vitro* testing procedures to better simulate *in vivo* conditions and thus improve the ability of the test to predict the outcome of ß-lactam therapy. These modifications will become increasingly important as clinically available metallo-β-lactamase inhibitors progress through development to differentiate their activity from our existing antimicrobial agents.

The major limitation of this study was the sample size of the observational, clinical study, which prohibited comparative assessment of outcomes between the MBL-active and non-MBL-active groups. This hinders inferences about effective therapeutic approaches as the study was not sufficiently powered to detect a difference in mortality between the 2 patient groups. Additionally, several imbalances in baseline and infection-related characteristics were noted (eg, bacteremia source and unit of admission), suggesting that the patients belonging to the MBL group had a higher acuity of illness at the start of treatment. SOFA scores were numerically higher in the MBL-active group, although the difference may not be clinically significant as SOFA scores ≤6 result in a similar percentage of patients having in-hospital mortality, at ≤10% [27]. Similarly, the non-MBL-active group had a higher comorbidity burden, as defined by a numerically higher Charlson Comorbidity Index. Moreover, the majority of patients in the non-MBL-active group were admitted in the first period of the NDM outbreak (2018–2019), while 52% of the MBL-active patients were admitted in 2020–2021, a reflection of the updated empiric antibiotic choices. Thus, the outcome of therapy may have been confounded by variation in patients’ characteristics as well as in time periods.

Our data suggest that the efficacy of carbapenems may be underestimated using current susceptibility testing methodologies and provide foundational support to help bridge the PK/PD relationships using MICs derived in low physiologic zinc conditions. Future studies should assess the zinc unbound concentration in infected patients’ plasma to better understand the interpatient variability and the influence of infection on zinc concentrations.

## Supplementary Data

Supplementary materials are available at *Open Forum Infectious Diseases* online. Consisting of data provided by the authors to benefit the reader, the posted materials are not copyedited and are the sole responsibility of the authors, so questions or comments should be addressed to the corresponding author.

## Supplementary Material

ofae228_Supplementary_Data

## References

[1] Chibabhai V, Nana T, Bosman N, Thomas T, Lowman W. Were all carbapenemases created equal? Treatment of NDM-producing extensively drug-resistant Enterobacteriaceae: a case report and literature review. Infection 2018; 46:1–13. 28916900 10.1007/s15010-017-1070-8

[2] Kohler PP, Volling C, Green K, Uleryk EM, Shah PS, McGeer A. Carbapenem resistance, initial antibiotic therapy, and mortality in *Klebsiella pneumoniae* bacteremia: a systematic review and meta-analysis. Infect Control Hosp Epidemiol 2017; 38:1319–28. 28950924 10.1017/ice.2017.197

[3] Zmarlicka MT, Nailor MD, Nicolau DP. Impact of the New Delhi metallo-beta-lactamase on beta-lactam antibiotics. Infect Drug Resist 2015; 8:297–309. 26345624 10.2147/IDR.S39186 PMC4554481

[4] Mura M, Longo B, Andreini R, et al Clinical outcomes in elderly patients with infections caused by NDM-producing *Klebsiella pneumoniae*: results from a real-life retrospective single center study in an endemic area. Intern Emerg Med 2023; 18:2261–9. 37698741 10.1007/s11739-023-03416-3

[5] Asempa TE, Abdelraouf K, Nicolau DP. Metallo-β-lactamase resistance in Enterobacteriaceae is an artefact of currently utilized antimicrobial susceptibility testing methods. J Antimicrob Chemother 2020; 75:997–1005. 31930305 10.1093/jac/dkz532

[6] MacVane SH, Crandon JL, Nichols WW, Nicolau DP. Unexpected *in vivo* activity of ceftazidime alone and in combination with avibactam against New Delhi metallo-β-lactamase-producing Enterobacteriaceae in a murine thigh infection model. Antimicrob Agents Chemother 2014; 58:7007–9. 25223998 10.1128/AAC.02662-14 PMC4249434

[7] Wiskirchen DE, Nordmann P, Crandon JL, Nicolau DP. Efficacy of humanized carbapenem exposures against New Delhi metallo-β-lactamase (NDM-1)-producing Enterobacteriaceae in a murine infection model. Antimicrob Agents Chemother 2013; 57:3936–40. 23733463 10.1128/AAC.00708-13 PMC3719754

[8] Kubicek-Sutherland JZ, Heithoff DM, Ersoy SC, et al Host-dependent induction of transient antibiotic resistance: a prelude to treatment failure. EBioMedicine 2015; 2:1169–78. 26501114 10.1016/j.ebiom.2015.08.012 PMC4588393

[9] Rex JH, Pfaller MA. Has antifungal susceptibility testing come of age? Clin Infect Dis 2002; 35:982–9. 12355386 10.1086/342384

[10] Boutzoukas AE, Komarow L, Chen L, et al International epidemiology of carbapenemase-producing *Escherichia coli*. Clin Infect Dis 2023; 77:499–509. 37154071 10.1093/cid/ciad288 PMC10444003

[11] Fuchs PC, Barry AL, Brown SD. Daptomycin susceptibility tests: interpretive criteria, quality control, and effect of calcium on *in vitro* tests. Diagn Microbiol Infect Dis 2000; 38:51–8. 11025184 10.1016/s0732-8893(00)00164-4

[12] Hackel MA, Tsuji M, Yamano Y, Echols R, Karlowsky JA, Sahm DF. *In vitro* activity of the siderophore cephalosporin, cefiderocol, against carbapenem-nonsusceptible and multidrug-resistant isolates of gram-negative bacilli collected worldwide in 2014 to 2016. Antimicrob Agents Chemother 2018; 62:e01968-17. 29158270 10.1128/AAC.01968-17 PMC5786755

[13] Cheng Z, Thomas PW, Ju L, et al Evolution of New Delhi metallo-β-lactamase (NDM) in the clinic: effects of NDM mutations on stability, zinc affinity, and mono-zinc activity. J Biol Chem 2018; 293:12606–18. 29909397 10.1074/jbc.RA118.003835 PMC6093243

[14] Abdelraouf K, Reyes S, Nicolau DP. The paradoxical *in vivo* activity of beta-lactams against metallo-beta-lactamase-producing Enterobacterales is not restricted to carbapenems. J Antimicrob Chemother 2021; 76:684–91. 33179050 10.1093/jac/dkaa467

[15] Falcone M, Tiseo G, Antonelli A, et al Clinical features and outcomes of bloodstream infections caused by New Delhi metallo-beta-lactamase-producing Enterobacterales during a regional outbreak. Open Forum Infect Dis 2020; 7:ofaa011. 32042848 10.1093/ofid/ofaa011 PMC7003035

[16] Abdelraouf K, Kim A, Krause KM, Nicolau DP. *In vivo* efficacy of plazomicin alone or in combination with meropenem or tigecycline against Enterobacteriaceae isolates exhibiting various resistance mechanisms in an immunocompetent murine septicemia model. Antimicrob Agents Chemother 2018; 62:e01074-18. 29866866 10.1128/AAC.01074-18 PMC6105799

[17] Clinical and Laboratory Standards Institute. Methods for Dilution Antimicrobial Susceptibility Tests for Bacteria That Grow Aerobically; Approved Standard. 11th ed. M07. Clinical and Laboratory Standards Institute; 2018.

[18] Clinical and Laboratory Standards Institute. Performance Standards for Antimicrobial Susceptibility Testing. 33rd ed. CLSI Supplement M100. Clinical and Laboratory Sciences Institute; 2023.

[19] Li C, Kuti JL, Nightingale CH, Nicolau DP. Population pharmacokinetic analysis and dosing regimen optimization of meropenem in adult patients. J Clin Pharmacol 2006; 46:1171–8. 16988206 10.1177/0091270006291035

[20] Trang M, Seroogy JD, Van Wart SA, et al Population pharmacokinetic analyses for plazomicin using pooled data from phase 1, 2, and 3 clinical studies. Antimicrob Agents Chemother 2019; 63:e02329–18. 30670433 10.1128/AAC.02329-18 PMC6496156

[21] Reyes S, Abdelraouf K, Nicolau DP. In vivo activity of human-simulated regimens of imipenem alone and in combination with relebactam against *Pseudomonas aeruginosa* in the murine thigh infection model. J Antimicrob Chemother 2020; 75:2197–205. 32386408 10.1093/jac/dkaa145

[22] Zasowski EJ, Bassetti M, Blasi F, et al A systematic review of the effect of delayed appropriate antibiotic treatment on the outcomes of patients with severe bacterial infections. Chest 2020; 158:929–38. 32446623 10.1016/j.chest.2020.03.087

[23] Lodise TP, Berger A, Altincatal A, et al Antimicrobial resistance or delayed appropriate therapy-does one influence outcomes more than the other among patients with serious infections due to carbapenem-resistant versus carbapenem-susceptible Enterobacteriaceae? Open Forum Infect Dis 2019; 6:ofz194. 31198817 10.1093/ofid/ofz194 PMC6546203

[24] Falcone M, Bassetti M, Tiseo G, et al Time to appropriate antibiotic therapy is a predictor of outcome in patients with bloodstream infection caused by KPC-producing *Klebsiella pneumoniae*. Crit Care 2020; 24:29. 32000834 10.1186/s13054-020-2742-9 PMC6993311

[25] Zhang Y, Kashikar A, Bush K. In vitro activity of plazomicin against β-lactamase-producing carbapenem-resistant Enterobacteriaceae (CRE). J Antimicrob Chemother 2017; 72:2792–5. 29091224 10.1093/jac/dkx261

[26] Nicolau DP . Pharmacokinetic and pharmacodynamic properties of meropenem. Clin Infect Dis 2008; 47(Suppl 1):S32–40. 18713048 10.1086/590064

[27] Vincent J-L, de Mendonca A, Cantraine F, et al Use of the SOFA score to assess the incidence of organ dysfunction/failure in intensive care units: results of a multicenter, prospective study. Crit Care Med 1998; 26:1793–800. 9824069 10.1097/00003246-199811000-00016

