# Supplementary material for: Deciphering the Efficacy of β-Lactams in the Face of Metallo-β-Lactamase-Derived Resistance in Enterobacterales: Supraphysiologic Zinc in the Broth Is the Culprit
Source: Open Forum Infect Dis. 2024 Apr 23;11(5):ofae228. doi: 10.1093/ofid/ofae228 (PMC11134298; doi:10.1093/ofid/ofae228)
Supplement: ofae228_Supplementary_Data [file ofae228_supplementary_data.docx]

## Supplemental Table S1: Antibiotic regimens received by the non-MBL active group

| **Patient ID** | **Empiric therapy** | **Definitive therapy** |
| --- | --- | --- |
| 71 | Meropenem 6 g over24 h | Ceftazidime/avibactam 2.5 g q12h; Aztreonam 2 g q8h |
| 72 | Meropenem 500 mg q8h | Tigecycline 50 mg q12h; Fosfomycin 4g q8h |
| 74 | Cefepime 2 g q12h; Meropenem 2 g q8h | Ceftazidime/avibactam 2.5 g q8h; aztreonam 2 g q8h |
| 75 | Meropenem 2 g q8h | Tigecycline 100 mg q12h; colistin 4.5 MU q12h; Ceftazidime/avibactam 2.5 g q8h |
| 76 | Piperacillin/tazobactam 4.5 q q8h; Meropenem 2 g q8h | Tigecycline 100 mg q12h; Meropenem 2 g q8h |
| 77 | Sulfamethoxazole/trimethoprim*; Piperacillin/tazobactam 4.5 g q8h; Meropenem 2 g q8h | Colistin 3 MU q12h; Meropenem 1 g q8h |
| 78 | Meropenem 1 g q8h | Colistin 4.5 MU q12h; Piperacillin/tazobactam 4.5 g q8h |
| 79 | Meropenem 1 g q8h | colistin 3 MU q8h; Fosfomycin 4 g q8h; Meropenem 1 g q8h |
| 80 | Meropenem 1 g q8h | Meropenem 1 g q8h; Tigecycline 50 mg q12h; Colistin 4.5 MU q12h |
| 81 | Piperacillin/tazobactam 2.25 gq8h; Meropenem 500 mg q6h | Meropenem 500 mg q6h; Tigecycline 50 mg q12h; Fosfomycin |
| 82 | Meropenem 2 g q8h | Colistin 4.5 MU q12h; Meropenem 2 g q8h; gentamicin |
| 83 | Imipenem/cilastatin 500 mg q8h | Colistin 4 MU q12h; Fosfomycin 4 g q6h |
| 84 | Imipenem/cilastatin 1 g q8h | Colistin 4 MU q8h; Tigecycline 50 mg q12h; Ceftazidime/avibactam 2.5 g q8h; Aztreonam 2 g q8h |
| 85 | Meropenem 1 g q8h | Ceftazidime/avibactam 2.5 g q8h; Aztreonam 2 g q8h; Tigecycline 100 mg q12h |
| 86 | Meropenem 2 g q8h | Ceftazidime/avibactam 2.5 g q8h; Aztreonam 2 g q8h; Tigecycline 100 mg q12h |
| 87 | Meropenem 2 g q8h | Ceftazidime/avibactam 2.5 g q8h; Aztreonam 2 g q8h |
| 88 | Meropenem 2 g q8h | Ceftazidime/avibactam 1.25 g q8h; Aztreonam 1 g q8h; Fosfomycin 3 g q8h |
| 89 | Meropenem 1 g q8h | Fosfomycin 4 g q8h |
| 90 | Meropenem 1 g q12h | Ceftazidime/avibactam 1.25 g q8h; Aztreonam 1 g q8h |
| 91 | Piperacillin/tazobactam 4.5 g q8h;Ceftazidime/avibactam 2.5 g q8h; Gentamicin* | Empiric continued |
| 92 | Ceftazidime/avibactam 1.25 g q8h | Ceftazidime/avibactam 2.5 g q8h; Aztreonam 2 g q8h |
| 93 | Meropenem 2 g q8h; Fosfomycin 4 g q8h* | Empiric continued |
| 94 | Ciprofloxacin 750 mg q12h*; Meropenem 1 g q8h | Empiric continued |
| 95 | Meropenem 1 g q12h | Empiric continued |
| 97 | Meropenem 1 g q8h | Empiric continued |
| 98 | Meropenem 2 g q8h | Ceftazidime/avibactam 2.5 g q8h; Aztreonam 2 g q8h |
| 99 | Ceftazidime/avibactam 1.25 g q8h | Colistin 3 MU q12h; fosfomycin 2 g q8h |
| 100 | Meropenem 2 g q8h | Ceftazidime/avibactam 2.5 g q8h; Aztreonam 2 g q8h |
| 101 | Meropenem 1 g q8h | Ceftazidime/avibactam 2.5 g q8h; Aztreonam 2 g q8h |

*Not active *in vitro.* The standard policy for meropenem infusion duration was 2 h.

## Supplemental Table S2: Zinc concentrations in CAMHB prior to and following treatment with Chelex as well as following zinc concentration adjustment

|  | **Replicate 1** | **Replicate 2** | **Replicate 3** | **Average ± Standard Deviation** |
| --- | --- | --- | --- | --- |
| **CAMHB** | 1148.1 | 1122.4 | 1109.1 | 1126.5 ± 19.8 |
| **Chelex-treated CAMHB** | 11.2 | 9.8 | 9.8 | 10.3 ± 0.8 |
| **Physiologic zinc concentration broth** | 54.8 | 54.1 | 55.1 | 54.7 ± 0.5 |
